# Uncertainty and enterprise export recovery in China: Does the market integration matter?

**DOI:** 10.1371/journal.pone.0309428

**Published:** 2024-12-23

**Authors:** Xiugang Zhu, Kangjuan Lv, Jingkai Gao

**Affiliations:** 1 School of Economics, Shanghai University, Shanghai, China; 2 SILC Business School, Shanghai University, Shanghai, China; 3 Xiamen University Tan Kah Kee College, Zhangzhou, China; Universiti Malaysia Sabah, MALAYSIA

## Abstract

Maintaining the stability of foreign trade in an uncertain environment is crucial to building a new development pattern. By combing through the existing literature, this paper analyzes the impact of economic policy uncertainty on enterprise export recovery from the perspective of market integration. Firstly, theoretical analysis shows that economic policy uncertainty is expected to attenuate enterprise export recovery levels by amplifying export transaction costs. Secondly, the escalation in market integration is anticipated to mitigate the attenuation above effect through the mechanism of "enhanced production efficiency", while simultaneously amplifying it through the mechanism of "intensified market competition". Based on the empirical test of China’s micro-level data, it is found that the rise of market integration generally alleviates the weakening effect of economic policy uncertainty on enterprise export resilience, and this mitigation effect is more obvious for high-efficiency enterprises; The test results of further mechanism analysis and heterogeneity analysis are also consistent with the logic of theoretical analysis.

## 1. Introduction

With frequent international trade disputes, rising anti-globalization trends, and escalating geopolitical conflicts, the global economy has been significantly impacted and faces increasing uncertainties. In light of the consensus on market integration, maintaining the resilience of China’s foreign trade in an uncertain environment is not only a crucial demand for stable economic development under the new development pattern but also an essential guarantee for enterprise survival and growth amidst shocks. It has become a common concern among scholars to elucidate the economic rationale behind stable foreign trade development under shocks and explore practical approaches to accelerate foreign export recovery while enhancing its resilience.

In contrast to developed countries, Chinese enterprises face the trade-off between "dual markets and dual resources" due to long-standing market segmentation and the separation of foreign trade [1–3]. Owing to higher market entry barriers and competitive pressures, inefficient enterprises are forced to engage in export-oriented trade with lower profit margins at the expense of markets [4]. Currently, promoting the integration of the market plays a crucial role in building a new development pattern that mutually reinforces both international cycles. The economies of scale resulting from the integration of the market contribute to dismantling institutional trade barriers between regions [5]. Therefore, when discussing the process of export recovery for Chinese enterprises in an uncertain environment, it is essential to consider the role of the market. Consequently, we need to address the following questions: How does uncertainty impact enterprise export recovery? What role does greater integration into the market play? Providing scientific answers to these inquiries not only helps clarify the microeconomic rationale behind enterprise export recovery amidst uncertainty but also enhances our understanding of the relationship between a vast market and foreign trade resilience.

With the increasing uncertainty, the existing literature lacks a consistent answer regarding how uncertainty precisely affects enterprise export recovery. On the one hand, uncertainty generally undergoes significant changes after shocks occur (such as Brexit, US-China trade frictions, etc.) and the specific impact of changes in existing uncertainty on enterprise export recovery remains rarely discussed. On the other hand, market integration has diverse effects on enterprise export trade based on their efficiency levels. Therefore, it is imperative to further investigate the influence of uncertainty on enterprise export recovery margin from the perspective of market integration.

Based on the existing literature, this paper expands upon the research topic to a certain extent. It integrates macro-level uncertainty, Meso-level market integration, and micro-level enterprise export recovery into a unified framework to analyze the impact of uncertainty on enterprise export recovery following shocks. Additionally, it categorizes enterprises based on their efficiency and explores the specific effects of market integration on different efficiency levels. The study confirms that improving market integration can enhance enterprise resilience in exporting. Furthermore, this positive moderating effect is more pronounced for efficient enterprises while inefficient export enterprises struggle to benefit from it. Therefore, this paper provides empirical evidence for the micro-level mechanism underlying the relationship between market integration, uncertainty, and enterprise export recovery.

The main innovations and contributions of this paper lie in four areas: (1) This paper introduces an innovative perspective by analyzing macro-level uncertainty, meso-level market integration, and micro-level enterprise exports in the same framework, providing a new research perspective. (2) Identifying positive and negative channels, and pioneeringly analyzes the impact of market integration on the two channels of uncertainty and enterprise export recovery from both positive and negative perspectives of "efficiency enhancement" and "competition intensification", and at the same time, this paper enriches the literature on the topic of uncertainty and export trade. At the same time, this paper enriches the literature on the topic of uncertainty and export trade. (3) Distinguishing the differences in efficiency, this paper focuses on the perspective of enterprises’ productivity differences to test the heterogeneous impact of market integration on the enterprise export recovery with different efficiencies under uncertainty and enriches the relevant literature studying the impact of domestic market integration on economic and trade development. (4) Offering policy recommendations, the paper’s conclusions have significant policy guidance that can help emerging market countries such as China to quickly recover their exports under uncertainty.

The subsequent content of this paper is organized as follows. The second section is the literature review, this part systematically sorts out the related theories of uncertainty, market integration, and enterprise export. The third section discusses the theoretical development and presents the hypothesis through theoretical analysis and formulation of research hypotheses. In the fourth section, we introduce the employed research methods, construct the model, and elaborate on data sources and processing techniques. The fifth section presents empirical results followed by an in-depth analysis of these findings. Finally, in the concluding section, we summarize the entire study and provide relevant policy recommendations.

## 2. Literature review

The literature relevant to the topic of this paper is focused on three main areas. This paper first examines the impact of uncertainty on enterprise export recovery after the occurrence of shocks, and thus, the first category of literature that we have sorted out in this paper mainly focuses on the topic of "Uncertainty and Enterprise Export Trade". Handley and Limao [6], and Al-Thaqeb et al. [7] have conducted comprehensive research reviews on the topic of "uncertainty and trade". Handley and Limao examined the effects of uncertainty in tariff policies on exports, aggregate prices, and trade welfare within the framework of a general equilibrium model [6]. Liu et al. demonstrated that the export implications of economic policy uncertainty are contingent upon trade patterns, and investigated its effects on particular industries. Their study revealed a notable deterrent effect of economic policy uncertainty on the exportation of electromechanical products [8]. Carballo et al. conducted a deeper investigation into the synergistic influences of policy interventions and economic shocks employing the sunk cost methodology [9]. Trinh et al. scrutinized the ramifications of uncertainty on enterprise investment efficiency. Their study illustrates that heightened uncertainty exacerbates financing constraints or induces enterprises to defer investment initiatives, consequently diminishing overall investment efficiency [10]. Alessandria et al. utilized an inventory-based model to elucidate the high-frequency intricacies of trade dynamics at the producer level or in the transmission of shocks. They contend that robust trade expansion ensues with heightened anticipated future uncertainty, a departure from prior literature findings [11]. Gervais discussed the relationship between uncertainty and outsourcing diversification [12]. Novy and Taylor delved deeper into investigating the influence of enterprises’ reactions to uncertainty on the overarching volatility of trade [13]. Lv Yue et al. conducted a comprehensive analysis of the trade risk transmission effects experienced by exporting enterprises during the financial crisis. They quantified the upstream degree at the Chinese enterprise level and validated the presence of the "long whip effect" hypothesis about value chain transmission during the crisis [14]. Qi Jianhong et al. found that escalating uncertainty reduces enterprise export frequency through the channels of trade costs and inventory costs [15]. Liu Qing et al. further investigated the country agglomeration behavior of heterogeneous exporting enterprises amidst uncertainty [16].

As global uncertainty continues to rise, scholarly attention has turned to issues concerning enterprise export recovery or export resilience following shocks. Naturally, literature of this nature is pertinent to the research at hand. He Canfei et al. [17], He Canfei, and Chen Tao [18] analyzed the "city-industry" export resilience from the perspective of the correlation between export products in Chinese cities. Liu Hui and Qi Jianhong [19], and Jiang Shuaishuai and Liu Hui [20], respectively explored the impact of diversification strategies and global value chain integration on the enterprise export resilience under external demand shocks. Dai et al. further discovered that, under the impact of the epidemic, agglomeration based on hometown relations is conducive to enhancing the resilience of enterprises [21]. Hu Zhaoling and Gao Xiaotong, taking the 2008 financial crisis as an example, studied the promoting effect of enterprise trade networks on the recovery of trade relationship exports by constructing enterprise self-trade network indicators and utilizing survival analysis [22].

The third category of literature relevant to this study focuses on analyzing the impact of the market and its degree of integration on export trade. Existing research suggested that introducing the assumption of rising marginal costs into classical heterogeneous enterprise trade models naturally leads enterprises to face substitution relationships between foreign markets [23, 24]. Moreover, financing constraints and the degree of market integration are also significant influencing factors that lead enterprises to balance between and foreign markets. This is mainly manifested as follows. On the one hand, the expansion of the market alleviates enterprises’ financing constraints and consequently promotes export trade [25]. On the other hand, the fragmentation of the market due to an imbalance in credit allocation also encourages inefficient enterprises to sacrifice sales in favor of exporting [1, 2, 26]. Sun Guofeng et al. argued that market integration can significantly reduce the degree of market segmentation in the local area, exhibiting a positive spatial spillover effect [27]. Wang Xiaobing et al. suggest that the agglomeration of high-tech industries imposes a single threshold level of market integration on the complexity of export technology [28]. Ma Zhaoliang et al. pointed out that the construction of market integration reinforces the promoting effect of structural competition in the banking industry on the export quality of the manufacturing industry [29].

Most of the existing studies are based on the theory of monopolistic competition in international trade and incorporates uncertainty into the research framework, further broadening the heterogeneous enterprises’ trade model, but it still fails to provide a consistent answer to the question of how uncertainty precisely affects enterprise exports. Therefore, this paper integrates macro-level uncertainty, Meso-level market integration, and micro-level enterprise export situations into a unified framework to analyze the effect of uncertainty on the recovery of enterprise exports following shocks, and it also explores the role of domestic market integration in this relationship. The combination of theoretical analysis and empirical research not only enriches the literature on the impact of uncertainty on enterprise exports but also expands the research area of this literature by introducing market integration into it.

## 3. Theoretical analyses and research hypotheses

### 3.1. Uncertainty and enterprise export recovery

Export recovery capability refers to the ability of economic entities to restore their economic development level to the pre-crisis level after a crisis occurs. It is one of the significant implications of export recovery. Additionally, export resilience also encompasses resistance during crisis occurrence and the capacity for self-renewal and repositioning after the crisis [30]. On the one hand, rising uncertainty increases the probability of business bankruptcy and moral hazards, exacerbates enterprises’ financing constraints, diminishes their competitive incentives and innovation drive, raises enterprises’ financing costs, and reduces their ability to save costs [31–33].On the other hand, escalating uncertainty raises the export threshold and engenders a "cleansing effect", forcing inefficient or smaller-scale enterprises to exit the market. This increases the difficulty for enterprises to acquire trade information and facilitate communication, while also raising costs across various aspects such as market research and relationship maintenance [15, 34, 35]. In summary, the escalation of uncertainty leads to a series of increased export transaction costs for enterprises, which is detrimental to the recovery of export enterprises from shocks. Based on this, Hypothesis 1 is proposed in this paper.

**Fig 1:**
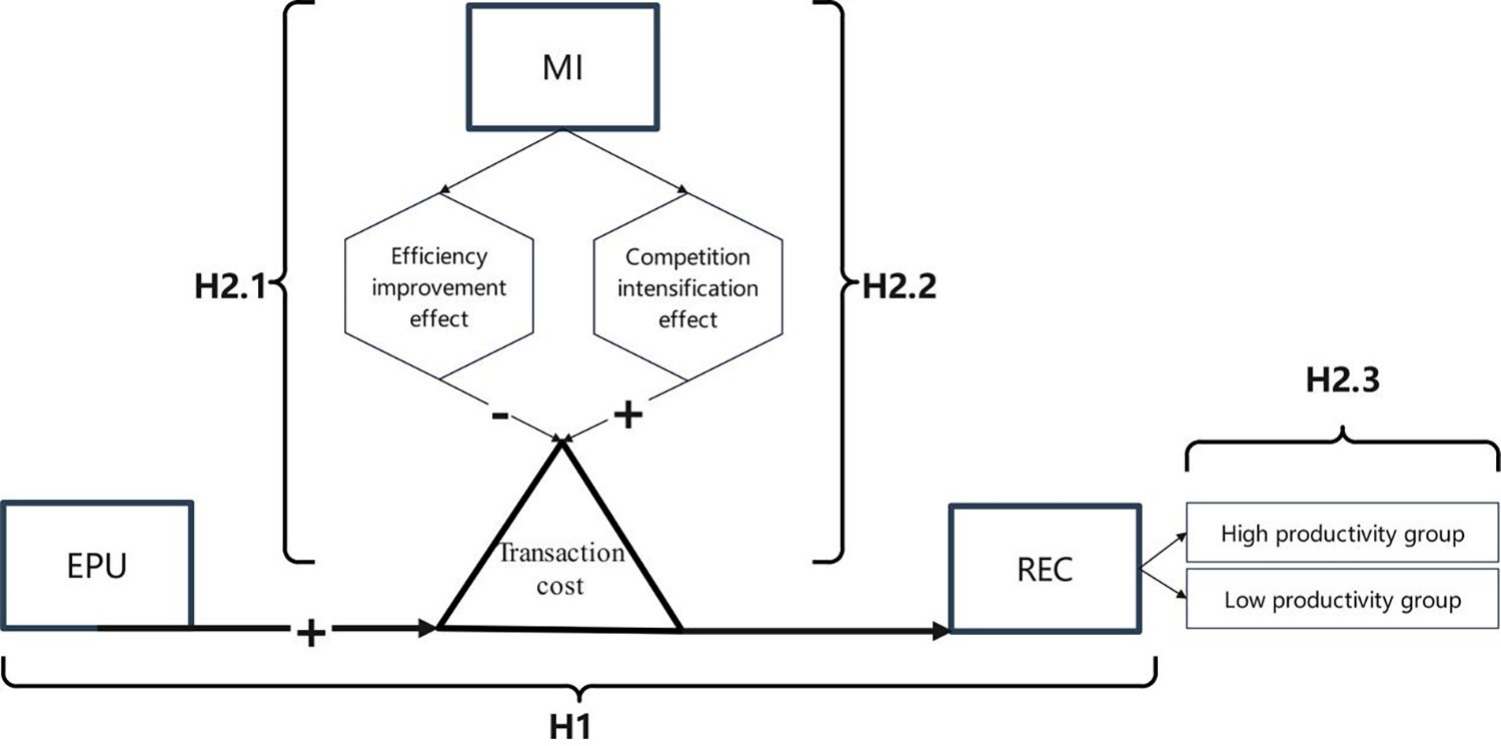
Theoretical mechanism. Note: EPU represents Economic Policy Uncertainty, REC represents Enterprise Export Recovery, and MI represents Market Integration.

**Hypothesis 1 (H1):** Holding other conditions constant, economic policy uncertainty stemming from shocks weakens enterprise export recovery capability (Fig 1).

### 3.2. The moderating effect of market integration

The impact of market integration on enterprise export recovery capability under uncertainty exhibits a dual effect.

On the one hand, some literature has found that market integration can directly have a positive impact on export trade by enhancing enterprises’ production scale and efficiency. Sheng and Mao found that market integration can help enterprises weaken trade barriers, reduce transaction costs, find better intermediate goods suppliers, and enhance the technological sophistication of export products [36]. Liu et al. argue that market integration accelerates the free flow of factors across regions, optimizes resource allocation in the market, fosters regional specialization, and further increases the technological sophistication of exports [37]. Lv et al. emphasize that market integration increases the value-added of exports for enterprises, leading to economies of scale benefits [14]. Liu found that considering the effects of foreign markets, market integration can improve the quality of exported products for enterprises [38]. In conclusion, the enhancement of market integration can expand enterprises’ market size, and improve resource allocation efficiency and specialization, thereby enhancing enterprises’ ability to cope with uncertainty, reducing transaction costs, and alleviating the weakening effect of economic policy uncertainty on enterprise export recovery capability. On the other hand, some literature suggests that market integration may also have adverse effects on export trade. Poncet argued that market integration disrupts local protectionism and reduces the international trade participation of enterprises [39]. Zhang et al. found that the deeper the level of market integration, the smaller the probability of local enterprises engaging in exports [40]. Gao similarly found that market integration reduces the cost for enterprises to enter markets in other regions, thereby decreasing their willingness to export [41]. In summary, the rise in the level of market integration can break down local protectionism and promote market competition, which may reduce enterprises’ average profits, and increase transaction costs such as market research, relationship maintenance, and information acquisition, thus exacerbating the weakening effect of uncertainty on enterprise export recovery capability.

Recent studies have begun to emphasize that market integration can have heterogeneous effects on export trade [42]. As described in classic literature such as Melitz [43], enterprises exhibit significant productivity differences in reality. Heterogeneous enterprises can expect vastly different benefits from the process of market integration: high-efficiency enterprises, with larger market shares and stronger market competitiveness, can derive greater efficiency improvement effects from the process of market integration and can mitigate the adverse effects of intensified competition. In contrast, low-efficiency enterprises face smaller market shares, which not only limit their positive effects but also expose them to more intense competition. Zhang et al. emphasize that market segmentation in the market makes it easier for low-efficiency enterprises to "abandon sales and choose exports" [3]. Therefore, as the level of market integration increases, low-efficiency enterprises are more inclined to exhibit weaker export recovery capability by tending towards "abandoning exports and shifting to sales." Based on this, Hypothesis 2 is proposed in this paper.

**Hypothesis 2 (H2):** In other unchanged conditions, **(H2.1)** the increase in the level of market integration will not only weaken the negative impact of uncertainty on enterprise export recovery capability through efficiency enhancement mechanisms, **(H2.2)** but also strengthen the negative impact of uncertainty on enterprise export recovery capability through intensified competition mechanisms. The net effect depends on the relative influence of these two mechanisms. **(H2.3)** The positive moderating effect of the increased level of market integration is more pronounced for high-efficiency enterprises (Fig 1).

## 4. Research design and data description

### 4.1. Model specification

To test the aforementioned theoretical hypotheses, this paper constructs the following econometric model:

$$REC_{ikjt}=\alpha_{0}+\alpha_{1}EPU_{\mathrm{jt}}+\alpha_{2}Z+\nu_{\mathrm{it}}+\nu_{\mathrm{ij}}+\varepsilon_{ikjt}$$ (1)

$$REC_{ikjt}=\beta_{0}+\beta_{1}EPU_{\mathrm{jt}}+\beta_{2}EPU_{\mathrm{jt}}\times MI_{kt-1}+\beta_{3}Z+\nu_{\mathrm{it}}+\nu_{\mathrm{ij}}+\xi_{ikjt}$$ (2)

Where subscript i represents the enterprise, k represents the province, j represents the destination, and t represents the year; REC represents the export recovery capability of the enterprise; EPU represents economic policy uncertainty; MI represents the level of market integration at the provincial level in China; EPU×MI represents their interaction term, economic policy uncertainty and market integration are respectively centralized, and then the interaction term is taken. For the convenience of expression, the original symbol is chosen in this paper; control variables Z include various control variables at different levels, as detailed in the variable description; *v*_*it*_ represents enterprise-year fixed effects; *v*_*ij*_ represents enterprise-destination fixed effects. The province is determined based on the first two digits of the customs enterprise code to ensure that there are no changes in the location of the enterprise; ε and ξ represent the random error terms.

### 4.2. Variable description

#### 4.2.1. Dependent variable

In this study, the export recovery capability of enterprises (REC) is defined at the enterprise-destination-year level, and following He Canfei and Chen Tao [16], the difference between the growth rate of export volume at the enterprise-destination-year level from 2009 to 2016 (G) and the corresponding growth rate in 2008 is used to measure the enterprise export recovery capability relative to the 2008 global financial crisis. A larger difference indicates a stronger export recovery capability of the enterprise. The calculation method for the midpoint growth rate is as Eq (3), and the calculation method for the enterprise export recovery capability is as Eq (4), where EXPORT represents the export amount, calculated as follows:

$$G_{ikjt}=2\times \frac{EXPORT_{ikjt}-EXPORT_{ikjt-1}}{EXPORT_{ikjt}+EXPORT_{ikjt-1}}$$ (3)

$${REC}_{ijkt}=G_{ijkt}{-G}_{ijk2008}$$ (4)

#### 4.2.2 Core explanatory variable

*4*.*2*.*2*.*1*. *Uncertainty*. This paper uses the monthly Economic Policy Uncertainty (EPU) index constructed by Baker et al. records the frequency of key terms such as "economy", "policy", and "uncertainty" appearing in mainstream newspapers in various countries, standardizes these data, and constructs a monthly EPU index, which includes EPU data from 23 countries or regions [44]. Referring to Rao Pingui et al. [31], in order to obtain the annual index, firstly, considering the most common mean value method, it is calculated using Eq (5), this method does not take into account the variability between different months and assumes that all the months are the same; secondly, considering that different time periods have variability, it is calculated using Eqs (6)–(8) and Table 1, this method takes into account the differences between the months and the quarters at the same time, assuming that different months have different weights. In particular, Table 1 shows the values of quarterly weights for different months. To obtain the annual index, methods proposed by Rao Pingui et al [31] and others are used to calculate the arithmetic mean and quarterly weighted average, as follows:

$$EPU_{jt}=\frac{\sum_{m=1}^{12}EPU_{jmt-1}}{12\times 100}$$ (5)

$$EPU2_{jt}=\frac{\sum_{q=1}^{4}\sum_{n=1}^{3}n\times EPU_{\mathrm{jmnqt}}}{4\times 6\times 100}$$ (6)

$$q=\{\begin{array}{l} 1\;m=1,2,3\\ 2\;m=4,5,6\\ 3\;m=7,8,9\\ 4\;m=10,11,12 \end{array}$$ (7)

$$n=\left\{ \begin{array}{c} 1\;m=1,4,7,10\\ 2\;m=2,5,8,11\\ 3\;m=3,6,9,12 \end{array}\right.$$ (8)

**Table 1:** Quarterly weight value.

		q				Quarterly weight
n		The first quarter	The second quarter	The third quarter	The fourth quarter	
	Order 1	January	April	July	October	1/6
	Order 2	February	May	August	November	2/6
	Order 3	March	June	September	December	3/6

In Eq (5), the simple average of the 12 monthly uncertainty indices is first derived, and then the annual uncertainty index is obtained by reducing the value by a factor of 100; In Eq (6)–(8) and Table 1, the quarterly weights are used to calculate the uncertainty index for each quarter, then a simple average of the uncertainty for the four quarters in the same year is derived, and finally the annual uncertainty index is obtained by reducing the value by a factor of 100. EPU indicates annual uncertainty, a larger EPU indicates higher uncertainty. Where m represents months, q represents quarters, and n represents the order of the months within each quarter.

*4*.*2*.*2*.*2 the level of market integration(MI)*. Market integration (MI). Inspired by Sheng Bin and Mao Qilin [40], first, this paper obtains the prices (P_kpt_) of eight continuously existing commodities (including food, tobacco and alcohol, clothing, housing, other goods and services, household equipment and services, health care and personal goods, transportation and communications, and entertainment, education and cultural goods and services) in 31 regions (including provinces, autonomous regions, and municipalities directly under the central government) in mainland China from the 2008–2016 China Statistical Yearbook, where k represents the region, p is for the commodity, and t is for the year. Next, the relative price ${\Delta P}_{k_{1}k_{2}pt}$ is obtained by finding the difference in price changes for the eight commodities according to Eq (9) below. Next, to avoid the price variance being affected by the order in which regions are placed, the absolute value of the relative price $\left|{\Delta P}_{k_{1}k_{2}pt}\right|$ is calculated. After that, in order to exclude the price changes caused by commodity heterogeneity, the paper uses the de-mean method for the treatment, and the new relative price ${\Delta Q}_{k_{1}k_{2}pt}$ is obtained according to the following Eq (10), where $\left|\bar{P_{pt}}\right|$ represents the mean value $\left|{\Delta P}_{k_{1}k_{2}pt}\right|$ of the relative prices between all combinations of regions, given year t and goods p; Then, the variance ${VAR\_\Delta Q}_{k_{1}k_{2}t}$ of ${\Delta Q}_{k_{1}k_{2}pt}$ of the relative price fluctuations of the eight commodities between every two regions is calculated according to the following Eq (11). In the next step, the relative price variance of all regional portfolios during the sample period is calculated according to Eq (12), and they are combined by region to calculate the market segmentation index VAR_ΔQ_st_ between each region and the rest of the country, where s represents a particular area combination, S represents the number of all area combinations; Finally, the market integration index is calculated for the sample according to Eq (13). In addition, in order to mitigate the endogeneity problem of the interaction term, this paper treats market integration (MI) lagged by one period.


$${\Delta P}_{k_{1}k_{2}pt}=ln(\frac{P_{k_{2}pt}}{P_{k_{2}pt-1}})-ln(\frac{P_{k_{1}pt}}{P_{k_{1}pt-1}})=\ln(\frac{P_{k_{2}pt}/P_{k_{2}pt-1}}{P_{k_{1}pt}/P_{k_{1}pt-1}})$$ (9)

$${\Delta Q}_{k_{1}k_{2}pt}=\left||\Delta P_{k_{1}k_{2}pt}|-|\bar{{\Delta P}_{pt}}|\right|$$ (10)

$${VAR\_\Delta Q}_{k_{1}k_{2}t}=var({\Delta Q}_{k_{1}k_{2}pt})$$ (11)

$${VAR\_\Delta Q}_{st=}\frac{\sum_{k_{1}\neq k_{2}}{VAR\_\Delta Q}_{k_{1}k_{2}t}}{S}$$ (12)

$$MI_{k_{1}t}=\sqrt{1/{VAR\_\Delta Q}_{st}}$$ (13)

#### 4.2.3. Control variables

The control variables in this study can be classified into two levels: (1) the enterprise-destination-year level: the logarithm of the number of HS6 categories, whether it is a bilateral trade relationship, whether it is solely processing trade, and whether it is mixed trade; (2) the destination-year level: the logarithm of regional gross product (GDP), the logarithm of population size, and the score of international economic freedom. In summary, this study involves control variables at two levels, and Table 2 reports the definition and descriptive statistics of the main variables.

**Table 2:** Definition and descriptive statistics of main variables.

Variables	Definition	Sample	Mean	St.dev	Min	Max
**REC**_**ikjt**_	Export recovery	1,116,035	-0.192	1.885	-4.000	4.000
**EPU**_**jt**_	Economic policy uncertainty	1,116,035	0.144	0.051	0.027	0.543
**MI**_**kt**_	Market integration	1,116,035	83.374	21.270	40.270	143.714
**HS6NUM**_**ikjt**_	Industry types	1,116,035	1.308	1.152	0.000	7.685
**EXIM**_**ikjt**_	Whether bilateral trade	1,116,035	-	-	0.000	1.000
**MODE1**_**ikjt**_	Whether processing trade	1,116,035	-	-	0.000	1.000
**MODE2**_**ikjt**_	Whether mixed trade	1,116,035	-	-	0.000	1.000
**GDP**_**jt**_	GDP of the destination	1,116,035	28.547	1.291	24.636	30.560
**POP**_**jt**_	Total population of the destination	1,116,035	18.013	1.255	15.244	21.004
**FREE**_**jt**_	Economic freedom of the destination	1,116,035	4.335	0.194	3.917	5.219

### 4.3. Sample selection

The sample data for this study is sourced from the China Customs database, Economic Policy Uncertainty (EPU) Index, China Statistical Yearbook, and other relevant databases detailed as follows: (1) In terms of data years, considering that the latest available year in the China Customs data is 2016 and the year of the shock is 2008, the sample period for this study is set from 2009 to 2016. (2) Concerning the China Customs database, firstly, the HS6 codes in the customs database are uniformly converted to the 1996 version. Secondly, based on the first 2 digits of the customs code, they are adjusted to the administrative division codes representing provinces. Each enterprise corresponds to only one province, and there are no changes in the geographical location of enterprises. Finally, the highly detailed data is aggregated to the enterprise-destination-year level, and relevant variables are calculated based on this. (3) Regarding the Economic Policy Uncertainty (EPU) Index, data for monthly EPU from 23 countries or regions were obtained, and annual EPU data were calculated using different weighting methods. (4) Regarding the China Statistical Yearbook, data on the month-on-month price indices of 8 products in 31 provinces or municipalities directly under the central government, excluding Hong Kong, Macao, and Taiwan, were obtained, and the level of market integration was calculated. Additionally, the study obtained CEP II gravity data, and economic freedom data from various regions worldwide. According to Brandt et al [32] and Yang Rudai [45], we used the LP method to obtain productivity data for each enterprise in 2007 from the China Industrial Enterprise Database. The LP method (Laspeyres production function method) is one of the most common methods for measuring total factor productivity (TFP). LP method is based on fixed assets, variable assets, labor, and other elements, through the linear programming model to estimate the production function, and then assess the enterprise’s production efficiency method, which can comprehensively measure the enterprise’s productivity level, including the resource utilization rate in the production process, the level of technology and economic efficiency and other factors, to provide important decision-making reference for the production and operation management of the enterprise. The equation for the LP method is as follows: ${TFP}_{LP}=\frac{Y}{\sum \alpha_{i}x_{i}+\sum \beta_{i}z_{i}}$, where Y represents the total output of the enterprise; *α*_*i*_ represents the coefficient of contribution of fixed assets; *x*_*i*_ represents the number of fixed assets; *β*_*i*_ represents the coefficient of contribution of variable assets; and *z*_*i*_ represents the number of variable assets. (The specific operation of obtaining enterprise productivity data from the China Industrial Enterprises Database through the LP method is detailed at https://feb.kuleuven.be/public/n07057/China/.)

## 5. Empirical results and analyses

### 5.1. Baseline test results

#### 5.1.1. Testing of hypothesis 1

In this section, the theoretical hypotheses are tested sequentially based on the econometric model. Table 3 presents the results of testing Hypothesis 1, where columns (1)-(2) examine the impact of economic policy uncertainty on enterprise export recovery capability, while columns (3)-(6) validate the underlying mechanisms of this impact. The dependent variable is the enterprise export recovery capability, with economic policy uncertainty as the core explanatory variable.

**Table 3:** Testing of Hypothesis 1.

	(1)	(2)	(3)	(4)	(5)	(6)
	Overall Sample		Group by distance		Group by the number of documents required for import	
			Shorter distance group	Longer distance group	Less file group	Multi file group
	REC	REC	REC	REC	REC	REC
**EPU**	-0.455***	-0.764***	-0.194	-2.566***	-0.057	-2.585***
	(-12.67)	(-21.37)	(-0.76)	(-17.76)	(-1.15)	(-21.12)
**Control variable**	NO	YES	YES	YES	YES	YES
**Enterprise-Year**	YES	YES	YES	YES	YES	YES
**Enterprise-Destination**	YES	YES	YES	YES	YES	YES
**Observation**	888,031	888,031	199,628	128,541	306,419	85,440
**Adjust-R2**	0.593	0.644	0.555	0.664	0.634	0.637

Note

***, **, and * denote p < 0.01, p < 0.05, and p < 0.1, respectively. Export transaction costs can be measured by using different percentages, import time, language similarity, religious proximity, and whether legal sources are the same, and the same results can be obtained.

Firstly, in columns (1)-(2) of Table 3, the first column controls for enterprise-year and enterprise-destination fixed effects without adding control variables. The coefficient of EPU is negative and statistically significant. Considering that the export recovery capability of enterprises may be influenced by factors at different levels, this study further includes control variables at the enterprise-destination-year and destination-year levels in the second column. The results show that the coefficient of EPU remains significantly negative. This indicates that holding other factors constant, economic policy uncertainty induced by shocks tends to weaken the average export recovery capability of enterprises, consistent with Hypothesis 1.

Furthermore, due to the lack of information on enterprise production and finance, it is difficult to directly measure export transaction costs. Therefore, this study uses two exogenous variables, namely, national distance and the number of documents required for destination import, as substitutes. Generally, the farther the distance and the greater the number of documents required, the higher the export transaction costs for enterprises. In columns (3)-(4) of Table 3, the first 30% of destination distances from China are considered as the short-distance group, while the last 30% are considered as the long-distance group. The results show that the coefficient of EPU in column (3) is negative but not significant, whereas in column (4), the coefficient of EPU is significantly negative, and the absolute value of the coefficient is larger than that in column (3). In columns (5)-(6) of Table 3, the first 30% of destination distances requiring import documents are considered as the few-document group, while the last 30% are considered as the many-document group. The results show that the coefficient of EPU in column (5) is negative but not significant, whereas in column (6), the coefficient of EPU is significantly negative, and the absolute value of the coefficient is larger than that in column (5). In summary, the farther the distance and the greater the number of documents required for import, the higher the export transaction costs. The weakening effect of economic policy uncertainty on enterprise export recovery capability becomes more pronounced, further supporting the economic mechanism implied by Hypothesis 1.

#### 5.1.2. Testing of hypothesis 2

Table 4 presents the test results for Hypothesis 2. Columns (1) to (2) examine the moderating effect of market integration on the attenuation effect identified in Hypothesis 1. Columns (3) to (4) further analyze the heterogeneity of the moderating effect based on the productivity differences among enterprises. The dependent variable in the regressions in Table 4 is the export recovery capacity of enterprises, with the core explanatory variables being economic policy uncertainty and its interaction with market integration.

**Table 4:** Testing of Hypothesis 1 and 2.3.

	(1)	(2)	(3)	(4)
	Overall Sample		Low-productivity group	High-productivity group
	REC	REC	REC	REC
**EPU**	-0.461***	-0.864***	-0.843***	-0.687***
	(-8.85)	(-17.38)	(-5.14)	(-4.94)
**EPU×MI**	0.000	0.005***	0.006	0.007**
	(0.20)	(3.51)	(1.08)	(1.98)
**Control variable**	NO	YES	YES	YES
**Enterprise-Year**	YES	YES	YES	YES
**Enterprise-Destination**	YES	YES	YES	YES
**Observation**	888,031	888,031	97,199	114,169
**Adjust-R2**	0.593	0.644	0.622	0.646

Note

***, **, and * denote p < 0.01, p < 0.05, and p < 0.1, respectively. Similar results can be obtained by grouping according to the top and bottom 30%, 25%, and 20% of productivity in 2007.

In the regression of column (1) in Table 4, controlling for enterprise-year and enterprise-destination fixed effects without additional control variables, the coefficient of EPU is significantly negative, while the coefficient of EPU×MI is insignificantly positive. Considering that enterprise export recovery capacity is influenced by various factors, in column (2), enterprise-destination-year and destination-year fixed effects are further included. The results show that the coefficient of EPU remains significantly negative, while the coefficient of EPU×MI becomes significantly positive. This indicates that overall, market integration can mitigate the weakening effect of economic policy uncertainty on enterprise export recovery capacity.

In columns (3) to (4) of Table 4, productivity is computed using the LP method in the industrial enterprise database. Based on the pre-shock year (2007) enterprise productivity, the sample is divided into two groups, with the bottom 30% of the industry categorized as the low-productivity group and the top 30% as the high-productivity group. In column (3), representing the low-productivity group, the coefficient of EPU×MI is positive but not significant. In column (4), representing the high-productivity group, the coefficient of EPU×MI is significantly positive, with a larger absolute value than in column (3). These results indicate that the positive moderating effect of market integration is more pronounced for high-efficiency enterprises, which is consistent with Hypothesis 2.

#### 5.1.3. Examination of the mechanism of hypothesis 2

Table 5 provides further examination of the theoretical mechanisms underlying Hypothesis 2. Columns (1) to (3) investigate the mechanism of efficiency improvement. Due to the difficulty in directly measuring the level of productivity at the enterprise-destination country level, the dependent variable in column (1) regression is the export value of core products. The HS6 corresponding to the maximum export value at the enterprise-destination-HS6-year level is selected as the core product at the enterprise-destination-year level, and the related export value is considered as the export value of core products at the enterprise-destination-year level. This is mainly because the mechanism of efficiency improvement will be reflected in the increase in the trade scale of core products. The dependent variable in the regression of column (2) is the export share, measured by the ratio of the export value at the enterprise-destination-year level to the export value at the enterprise-year level. This is mainly because the mechanism of efficiency improvement will also be reflected in the increase in the scale of foreign markets. Using this indicator can eliminate the influence of changes in the market scale and focus on analyzing the relative impact of market integration on specific export market scales. In column (3) regression, the dependent variable is the general trade export share, calculated by dividing the general trade export value at the enterprise-destination-year level by the total export value at the enterprise-destination-year level. Since general trade requires higher fixed costs compared to processing trade, it can be considered as a form of trade upgrading [4]. According to the logic of Hypothesis 2, market integration will expand the market scale, improve transaction efficiency, and increase the export value of core products, the export share of specific destination markets, and the export share of general trade.

**Table 5:** Examination of the mechanism of Hypothesis 2.1 and 2.2.

	(1)	(2)	(3)	(4)	(5)
	Efficiency improvement mechanism			Competition intensification mechanism	
	Mainproduct_value	Exportvalue_share	Exportmode_share	Export_price	Varieties_num*
**EPU**	-0.452***	-0.084***	-0.048***	47.522***	0.832***
	(-6.82)	(-18.42)	(-12.51)	(2.86)	(26.63)
**EPU×MI**	0.016***	0.002***	0.001***	-0.922**	-0.011***
	(8.38)	(13.19)	(8.28)	(-2.20)	(-12.34)
**Control variable**	YES	YES	YES	YES	YES
**Enterprise-Year**	YES	YES	YES	YES	YES
**Enterprise-Destination**	YES	YES	YES	YES	YES
**Observation**	888,031	888,031	888,031	804,002	888,031
**Adjust-R2**	0.668	0.618	0.532	0.545	0.715

Note

***, **, and * denote p < 0.01, p < 0.05, and p < 0.1, respectively. Considering the similarity in meaning between HS6NUM and Varieties_num, the variable HS6NUM is removed from the regression in column (5) of Table 5.

Columns (4) to (5) of Table 5 examine the mechanism of intensified competition. According to the analysis of Mayer et al. [46] and other literature, the competitive effect brought about by the increase in market scale can manifest as enterprises reducing the variety of products, focusing on producing core products to maintain market competitiveness. Therefore, the dependent variable in the regression of column (4) is the number of export product categories at the enterprise-destination-year level. Additionally, following the insights from Melitz and Ottaviano [47], when market competition intensifies, the average price level of exported products of enterprises tends to decrease. Hence, the export price at the enterprise-destination-year level is selected as the dependent variable in the regression of column (5). The core explanatory variables in the regressions of Table 5 are all the interaction terms between economic policy uncertainty and market integration.

Columns (1) to (3) of Table 5 indicate that the coefficient of EPU is significantly negative, while the coefficient of EPU×MI is significantly positive. This suggests that market integration can alleviate the inhibitory effect of uncertainty on core product export volume, enterprise export share, and general trade export share, thereby enhancing export recovery capability. Columns (4) and (5) reveal that the coefficient of EPU is significantly positive, while the coefficient of EPU×MI is significantly negative. This indicates that an increase in EPU promotes product diversification in exports and raises the average export price level for enterprises. However, the competitive effect of market integration weakens these effects, thereby weakening the export recovery capability of enterprises.

### 5.2 Endogeneity issues and robustness test

#### 5.2.1 Sample selection problem and treatment

Considering that the benchmark sample includes certain special cases, and the export resilience of enterprises in these cases may be deeply influenced by specific factors such as economic policies, rather than directly affected by the uncertainty of economic policies and the degree of market integration. This paper excludes the following special cases: (1) Excluding samples from free trade ports such as Singapore and Hong Kong, which are often considered tax havens rather than ultimate export destinations; (2) Excluding samples of trade intermediaries, as there are cases of cross-border exports by export intermediaries; (3) Excluding samples of processing trade, as processing trade is embedded in the international value chain cycle, detached from the production cycle, and characterized by dual foreign orientation. Table 6 reports the relevant regression results, with columns (1)-(2) showing regressions excluding free trade port samples; columns (3)-(4) showing regressions excluding trade intermediary samples; columns (5)-(6) showing regressions excluding foreign-funded enterprise samples; and columns (7)-(8) showing regressions excluding processing trade samples. As shown in Table 6, the coefficient of Economic Policy Uncertainty (EPU) is significantly negative, while the coefficient of the interaction term EPU × MI is significantly positive, indicating that the regression results remain consistent with the preceding analysis.

**Table 6:** Considering the impact of regression sample selection range.

	(1)	(2)	(3)	(4)	(5)	(6)
	Eliminate trade free ports		Eliminate trade middlemen		Eliminate trade processing samples	
	REC	REC	REC	REC	REC	REC
**EPU**	-0.846***	-1.028***	-0.654***	-0.733***	-0.757***	-0.832***
	(-23.27)	(-19.88)	(-13.48)	(-10.71)	(-20.36)	(-16.09)
**EPU×MI**		0.009***		0.004*		0.004**
		(5.98)		(1.95)		(2.53)
**Control variable**	YES	YES	YES	YES	YES	YES
**Enterprise-Year**	YES	YES	YES	YES	YES	YES
**Enterprise-Destination**	YES	YES	YES	YES	YES	YES
**Observation**	762,183	762,183	527,481	527,481	787,275	787,275
**Adjust-R2**	0.804	0.804	0.801	0.801	0.802	0.802

Note

***, **, and * denote p < 0.01, p < 0.05, and p < 0.1, respectively. Utilizing indicators such as incremental export value and its recovery, month-on-month growth rate of recovery, etc., yields consistent results. Regression results with only the inclusion of EPU also align with the baseline. Supplementary results are available upon request.

#### 5.2.2 Measurement error problem and treatment

Firstly, consider the influence of measurement errors in the dependent variable. On the one hand, given the abstraction of the concept of export resilience and the diversity of indicators, inspired by He Canfei and Chen Tao [18], this study sequentially adopts the following four indicators as alternative measures of enterprise export resilience: incremental export value and its recovery compared to 2008, midpoint growth rate, and month-on-month growth rate. Among them, although the incremental export value and growth rate do not explicitly represent "recovery," they intuitively reflect the competitive strength of export enterprises and the trend of export changes. On the other hand, the self-renewal ability of enterprises after experiencing shocks can also reflect their export resilience. This study measures whether the types of export products of enterprises have changed and the export value of new export products. Changes in the types of export products and the increase in the export value of new products both reflect the improvement of enterprises’ self-renewal capabilities. The measurement standard for whether the types of export products of enterprises have changed is defined as follows: when the types of products exported by enterprises to a specific destination in year t are completely consistent with those in year t-1, the value is 0; otherwise, the value is 1. The export value of new export products is measured by comparing the export value of products newly exported by enterprises to a specific destination in a specific year with that of the previous year at the enterprise-destination-year level. In general, promoting changes in the types of products exported by enterprises and increasing the value of exports of new products indicate that exporting enterprises have a strong capacity for self-renewal, which is conducive to the recovery of enterprises from shocks. Table 7 reports the relevant regression results, where columns (1)-(4) represent regressions with the dependent variables being export value, recovery amount of export value, midpoint growth rate, and month-on-month growth rate, respectively; columns (5) and (6) represent the dependent variables being new product export value and the variable indicating whether there has been a change in product types. From Table 7, it can be observed that the coefficient of Economic Policy Uncertainty (EPU) is significantly negative, and the coefficient of the interaction term EPU × MI is significantly positive, indicating the robustness of the regression results.

**Table 7:** Consider the effect of the measurement error of the explained variable.

	(1)	(2)	(3)	(4)	(5)	(6)
	Recovery after shock				Self-renewal capacity after shocks	
	Value	Value_REC	Grow_1	Grow_2	Value_New	Vars_Change
EPU	-0.379***	-0.379***	-0.864***	-12,682.340*	-0.993***	-0.095***
	(-6.07)	(-6.07)	(-17.38)	(-1.75)	(-9.38)	(-7.90)
EPU×MI	0.015***	0.015***	0.005***	434.495*	0.006**	0.001***
	(8.24)	(8.24)	(3.51)	(1.74)	(2.07)	(3.70)
**Control variable**	YES	YES	YES	YES	YES	YES
**Enterprise-Year**	YES	YES	YES	YES	YES	YES
**Enterprise-Destination**	YES	YES	YES	YES	YES	YES
**Observation**	888,031	888,031	888,031	888,031	589,340	888,031
**Adjust-R2**	0.728	0.804	0.040	0.178	0.533	0.469

Note

Tests using indicators such as the incremental export value and its recovery and the recovery of the chain growth rate were able to obtain the same results;The results of regressions that only put in a single EPU term are also consistent with the benchmark, if needed, on request.

On the other hand, consider the influence of measurement errors in the core explanatory variables. On one hand, as the frequency of uncertainty keywords is higher during major events or conferences and lower during regular periods, text-based measurements of uncertainty exhibit quarterly patterns. Therefore, this study further calculates annual economic policy uncertainty using a quarterly weighted average method. On the other hand, there is no unique method or indicator for calculating the level of market integration. This study utilizes the Marketization Index developed by Fan Gang to measure the level of market integration and conducts robustness tests. Additionally, considering endogeneity issues, this study also examines the exogenous MI index from 2008. Table 8 reports the relevant regression results. Columns (1)-(2) replace the EPU calculation method with quarterly weighted EPU, while maintaining the MI calculation method consistent with the baseline. Column (3) replaces the MI calculation with the Fan Gang Marketization Index while keeping the EPU calculation consistent with the baseline. Column (4) replaces both the EPU and MI calculations with quarterly weighted EPU and the Fan Gang Marketization Index, respectively. From Table 8, it can be observed that the coefficient of EPU is significantly negative, and the coefficient of the interaction term EPU × MI is significantly positive, indicating the robustness of the regression results.

**Table 8:** Considering the impact of measurement errors in the core explanatory variables.

	(1)	(2)	(2)	(3)
	Quarter-weighted EPU indicators		Fan Gang MI indicators	Quarter-weighted EPU and Fan Gang MI
	REC	REC	REC	REC
**EPU**	-81.863***	-93.111***	-0.948***	-100.717***
	(-22.40)	(-18.61)	(-19.25)	(-20.28)
**EPU×MI**		0.567***	0.178***	18.560***
		(4.04)	(6.47)	(6.71)
**Control variable**	YES	YES	YES	YES
**Enterprise-Year**	YES	YES	YES	YES
**Enterprise-Destination**	YES	YES	YES	YES
**Observation**	888,031	888,031	888,031	888,031
**Adjust-R2**	0.644	0.644	0.644	0.644

Note

***, **, and * denote p < 0.01, p < 0.05, and p < 0.1, respectively.

#### 5.2.3 Issues on omitted variables

To mitigate the issue of omitted variables, this study addresses it in two ways: firstly, by further adding destination-year level control variables on top of the existing control variables, including whether the country joined the WTO and the level of national governance. The regression results are shown in Table 9, columns (1)-(2). Secondly, to eliminate the influence of regional and industrial factors such as industrial agglomeration on the research results, this study further controls for province-industry (HS4)-year fixed effects. The regression results are presented in Table 9, columns (3)-(4). From Table 9, it can be observed that the regression results remain robust.

**Table 9:** Considering the impact of omitted variables.

	(1)	(2)	(3)	(4)
	Increase control variables		Increase the fixed effect	
	REC	REC	REC	REC
**EPU**	-0.618***	-0.670***	-0.779***	-0.905***
	(-17.22)	(-13.49)	(-20.99)	(-17.41)
**EPU×MI**		0.003*		0.006***
		(1.84)		(4.17)
**Original control variable**	YES	YES	YES	YES
**Enterprise-Year**	YES	YES	YES	YES
**Enterprise-Destination**	YES	YES	YES	YES
**Increase control variables**	YES	YES	NO	NO
**Province-industry-year**	NO	NO	YES	YES
**Observation**	888,031	888,031	861,377	861,377
**Adjust-R2**	0.645	0.645	0.646	0.646

Note

***, **, and * denote p < 0.01, p < 0.05, and p < 0.1, respectively. Add the control variables, including whether to join the WTO and the national governance level.

#### 5.2.4 Shock year problem and treatment

On the one hand, considering that the financial crisis erupted at the end of 2007, the baseline of this study set the shock year as 2008, which may lead to bias. Thus, this study replaces the shock year with 2007 to recalculate enterprise export resilience. On the other hand, after the outbreak of the financial crisis, due to the time needed for the transmission of economic crises, the response of economic entities, and the completion of old orders, the actual impact of the financial crisis may be delayed. Therefore, this study replaces the shock year with 2009 to reevaluate enterprise export resilience.

Table 10, columns (1)-(2), set the shock year as 2007, while columns (3)-(4) replace the shock year with 2009. Table 10 reports the relevant regression results, where the coefficient of EPU remains significantly negative, and the interaction term EPU × MI remains significantly positive. This indicates that the selection range of the shock year does not affect the empirical test conclusions.

**Table 10:** Consider the impact of the range of impact years selected.

	(1)	(2)	(3)	(4)
	Shock year with 2007		Shock year with 2009	
	REC	REC	ERC	REC
**EPU**	-0.673***	-0.890***	-0.700***	-0.806***
	(-20.35)	(-18.99)	(-19.53)	(-16.70)
**EPU×MI**		0.010***		0.005***
		(7.87)		(3.75)
**Control variable**	YES	YES	YES	YES
**Enterprise-Year**	YES	YES	YES	YES
**Enterprise-Destination**	YES	YES	YES	YES
**Observation**	903,489	903,489	753,420	753,420
**Adjust-R2**	0.660	0.660	0.673	0.673

Note

***, **, and * denote p < 0.01, p < 0.05, and p < 0.1, respectively.

#### 5.2.5 Other endogeneity issues and treatment

Economic policy uncertainty is a macro-level variable at the destination-year level, primarily influenced by changes in the international situation and destination trade policies. Market integration is a Meso-level variable at the province-year level, mainly affected by economic policies and provincial trade development policies, while enterprise export resilience is a micro-level variable at the enterprise-destination-year level. On one hand, micro-level enterprise export resilience is unlikely to exert a significant impact on macro-level or Meso-level variables, and Meso-level market integration is also unlikely to sufficiently influence macro-level economic policy uncertainty, especially considering that they measure different entities across countries or regions. On the other hand, economic policy uncertainty measures the uncertainty of destination markets, while market integration measures the economic development of provinces. The relationship between the two is not closely related. hence, economic policy uncertainty in international destinations is insufficient to affect the level of market integration in a province in China.

Additionally, this study lags the market integration indicator by one period to further alleviate endogeneity issues. To further examine whether there is a high correlation between economic policy uncertainty and market integration, and to address potential severe endogeneity issues arising from mutual effects in the regression, this study tests the relationship between economic policy uncertainty and market integration. The results, as shown in Table 11, indicate an extremely weak correlation between EPU and the degree of MI.

**Table 11:** Test the relationship between EPU and MI.

	EPU	MI
**EPU**	1	0.0361***
**MI**	0.0936***	1

Note

***, **, and * denote p < 0.01, p < 0.05, and p < 0.1, respectively. The Pearson test is in the lower left corner and the Spearman test is in the upper right corner.

### 5.3 Heterogeneity test results

#### 5.3.1 Heterogeneity testing based on enterprise geographic location

Market segmentation incentivizes distortions in enterprise exports, and the magnitude of this effect is negatively correlated with export trade costs [48]. Compared to the inland regions, border regions are adjacent to Central Asia and not far from European regions, while coastal regions are close to ports, allowing for quick and convenient access to various parts of the world via sea transport. Therefore, export transaction costs for enterprises in inland regions are much higher than those for enterprises in border and coastal regions. According to the theoretical logic analyzed in this study, market integration is more likely to alleviate the weakening effect of economic policy uncertainty on export resilience for enterprises in border and coastal regions. To verify this conclusion, this study divides the sample into inland region samples and border/coastal region samples. The results, as shown in columns (1)-(2) of Table 12, indicate that in the regression using inland region samples (column 1), the coefficient of EPU × MI is positive but not significant, whereas in the regression using border/coastal region samples (column 2), the coefficient of EPU × MI is significantly positive. Compared to enterprises in inland regions, market integration has a more pronounced alleviating effect on enterprises in border and coastal regions.

**Table 12:** Heterogeneity test results based on enterprises’ geographic location and industry level of financing constraints.

	(1)	(2)	(3)	(4)	(5)	(6)
	Location of the enterprise		Industry financing constraint level			
	Internal area	Border coastal area	Low external dependence	High external dependence	Low liquidity demand	High liquidity demand
	REC	REC	REC	REC	REC	REC
**EPU**	-0.798***	-0.869***	-0.888***	-0.748***	-0.811***	-0.818***
	(-4.75)	(-16.64)	(-12.22)	(-9.18)	(-11.02)	(-10.22)
**EPU×MI**	0.007	0.005***	0.002	0.007***	0.003	0.007***
	(1.61)	(3.23)	(1.10)	(3.18)	(1.56)	(3.03)
**Control variable**	YES	YES	YES	YES	YES	YES
**Enterprise-Year**	YES	YES	YES	YES	YES	YES
**Enterprise-Destination**	YES	YES	YES	YES	YES	YES
**Observation**	64,402	823,629	396,988	369,596	418,566	347,374
**Adjust-R2**	0.624	0.645	0.647	0.636	0.640	0.642

Note

***, **, and * denote p < 0.01, p < 0.05, and p < 0.1, respectively.

#### 5.3.2 Heterogeneity testing based on industry financing constraint levels

Kroszner et al. calculated liquidity constraint indicators for listed companies based on U.S. data from 1980 to 1999 [49]. Among them, there are 24 data points for financial fragility at the ISIC 3-digit level and nine data points for financial fragility at the ISIC 4-digit level. This study utilizes these data to measure external financing dependence and liquidity demand in industries. ISIC 3-digit codes are converted to HS6 codes, and relevant data are matched accordingly. The higher the external financing dependence and liquidity demand in an industry, the greater the export financing pressure faced by enterprises, and thus the greater the financing constraints and export difficulty constraints. According to the theoretical logic analyzed in this study, market integration is more likely to alleviate the weakening effect of economic policy uncertainty on export resilience for enterprises in industries with high external financing dependence or high liquidity demand. To verify this conclusion, this study divides the sample into high and low external financing dependence samples and high and low liquidity demand samples. The results, as shown in columns (3)-(6) of Table 12, indicate that in the regression using the low external financing dependence sample (column 3), the coefficient of EPU × MI is positive but not significant. In contrast, in the regression using the high external financing dependence sample (column 4), the coefficient of EPU × MI is significantly positive. Similarly, in the regression using the high liquidity demand sample (column 5), the coefficient of EPU × MI is positive but not significant, while in the regression using the high liquidity demand sample (column 6), the coefficient of EPU × MI is significantly positive. In summary, compared to the low external financing dependence sample and low liquidity demand sample, the alleviating effect of market integration is more pronounced in the high external financing dependence sample and high liquidity demand sample.

## 6. Conclusions and policy recommendations

Against the backdrop of rising global uncertainty, improving the sustainable export capacity of enterprises is crucial for stabilizing foreign trade. Departing from the perspective of market integration, this paper investigates the impact of economic policy uncertainty induced by shocks on enterprise export recovery capability. Theoretical analysis suggests that economic policy uncertainty resulting from shocks weakens enterprise export recovery capability through the channel of export transaction costs. Market integration expands the market size faced by enterprises, which, on the one hand, mitigates the above weakening effect by enhancing transaction efficiency, with this effect being more pronounced for high-productivity. On the other hand, it amplifies the above weakening effect by intensifying market competition, with this effect being more pronounced for low-productivity enterprises. Empirical results indicate that the moderating effect of the level of market integration is overall positive, and this effect is more significant for high-efficiency enterprises.

The policy implications of the conclusions of this study are reflected in two aspects: firstly, enhancing the level of market integration, coordinating internal and external efforts, and magnifying the effect of "transaction efficiency improvement". According to the research findings, market integration is generally conducive to enhancing enterprise export recovery capability under uncertainty. Therefore, it is imperative to actively build a national-level comprehensive open platform, optimize the mechanism for sharing trade information, enhance the level of integration of product and factor markets, and further unleash the positive effects of market scale, thereby weakening the adverse effects of information asymmetry caused by uncertainty on foreign trade enterprises, enhancing enterprise export recovery capability, and promoting the virtuous interaction between and international cycles. Secondly, precision formulation of differentiated support policies is essential to maximize benefits and minimize risks, alleviating the negative impacts brought by the "intensification of competition effect". As mentioned earlier, market integration enhances enterprise export recovery capability by optimizing multiple export margins. However, due to lower market share and intense competition resulting from market integration, low-efficiency export enterprises struggle to benefit. Therefore, while enhancing the level of market integration, the government should formulate targeted differentiated support policies to alleviate the pressure on the survival of low-efficiency enterprises under uncertainty, thereby stabilizing foreign trade and employment.

## Supporting information

S1 Data (RAR)

## References

[1] Zhu X, Jin X, Luo D. Market segmentation and the expansion of China’s export[J]. Economic Research Journal, 2005, 12: 68–76.

[2] Liu Q, Gui J J, Cheng L. Trade policy uncertainty and enterprises export dependency: from the perspective of domestic product market integration[J]. Finance and Trade Research,2020,31(09):1–15+110.

[3] Zhang X L, Cheng L, Liu Q. The linkage between export and domestic sale[J]. Finance & Trade Economics,2021,42(01):136–150.

[4] Liu Q, Cheng L, Shao Z, et al. Credit constraints, export mode and trade upgrading[J]. Economic Research Journal, 2017, 52(5): 75–88.

[5] Chen T, Yan Z X. Strengthening effect of scale economics in domestic market integration: from the perspective of reducing institutional trade frictions[J]. Journal of Quantitative & Technological Economics, 2024,41(04):5–25. doi: 10.13653/j.cnki.jqte.20240226.002

[6] Handley K, Limao N. Policy uncertainty, trade, and welfare: Theory and evidence for China and the United States[J]. American Economic Review, 2017, 107(9): 2731–83.

[7] Al-Thaqeb, Saud Asaad, and Barrak Ghanim Algharabali. "Economic policy uncertainty: A literature review." The Journal of Economic Asymmetries 20 (2019): e00133.

[8] Liu D, Zhu X, Yu H. Economic policy uncertainty, intra-industry trade, and China’s mechanical and electrical product exports[J]. Plos one, 2024, 19(1): e0290866. 38236940 10.1371/journal.pone.0290866 PMC10795980

[9] Carballo, Jeronimo, Handley Kyle, and Nuno Limão. Economic and policy uncertainty: Export dynamics and the value of agreements. No. w24368. National Bureau of Economic Research, 2018.

[10] Trinh N. Economic Policy Uncertainty and Corporate Investment Efficiency: Evidence from Australian Energy Companies[J]. International Journal of Energy Economics and Policy, 2024, 14(1): 53.

[11] Alessandria George A., Khan Shafaat Y., and Khederlarian Armen. Taking stock of trade policy uncertainty: evidence from China’s pre-WTO accession. No. w25965. National Bureau of Economic Research, 2019.

[12] Gervais Antoine. "Global sourcing under uncertainty." Canadian Journal of Economics/Revue canadienne d’économique 54.3 (2021): 1103–1135.

[13] Novy Dennis, and Taylor Alan M. "Trade and uncertainty." Review of Economics and Statistics 102.4 (2020): 749–765.

[14] Lv Y, Sheng B, Lv Y. Does market fragmentation curb firms’ DVAR in China[J]. Chin Ind Econ, 2018, 5: 5–23.

[15] Qi J H, Yi D, Liu H. Does economic policy uncertainty affect firms’ export decision? A study from the perspective of export frequency[J]. Journal of Financial Research,2020(05):95–113.

[16] Liu Q, Sun J, Su L M. Agglomeration by export-destination, uncertainty and export behavior of heterogeneous firms[J]. Journal of International Trade,2020(01):67–81.

[17] He C F, Li Z F, Chen H H. Cross-border mergers and acquisitions of Chinese enterprises under the influence of regional integration and institutional distance[J]. Progress in Geography,2019,38(10):1501–1513.

[18] He C F, Chen T. External demand shocks, related variety and resilience of export[J]. China Industrial Economics, 2019, 7: 61–80.

[19] Liu H, Qi J. Can diversification strategy enhance export resilience of the enterprises[J]. Int. Econ. Trade Res, 2021, 12: 4–19.

[20] Jiang S S, Liu H. The "double-edged sword" effect of embedding location in GVC on enterprise’s export resilience under crisis[J]. International Business,2021,(01):1–17. doi: 10.13509/j.cnki.ib.2021.01.001

[21] Dai R, Mookherjee D, Quan Y, et al. Industrial Clusters, Networks and Resilience to the Covid-19 Shock in China[J]. Journal of Economic Behavior & Organization, 2021, 183(3). doi: 10.1016/j.jebo.2021.01.017

[22] Hu Z L, Gao X T. Influence of the corporate trade network on the resumption of exports[J]. The Journal of World Economy,2022,45(05):113–136. doi: 10.19985/j.cnki.cassjwe.2022.05.004

[23] Feng L, Li Z, Swenson D L. Trade policy uncertainty and exports: Evidence from China’s WTO accession[J]. Journal of International Economics, 2017, 106: 20–36.

[24] Almunia Miguel, et al. "Venting out: Exports during a domestic slump." American Economic Review 111.11 (2021): 3611–3662.

[25] Erbahar Aksel. "Two worlds apart? Export demand shocks and domestic sales." Review of World Economics 156.2 (2020): 313–342.

[26] Manova Kalina, and Yu Zhihong. "How firms export: Processing vs. ordinary trade with financial frictions." Journal of International Economics 100 (2016): 120–137.

[27] Sun G F, Zou X Y, Wang H L. Research on the impact of opening up on the construction of a unified national market: based on market segmentation in China[J]. China Soft Science,2024(01):99–109.

[28] Wang X B, Liu F R. Research on the threshold effect of high-tech industry agglomeration on export technological complexity from the perspective of constructing a unified large market[J]. China Journal of Commerce,2024(01):16–20.

[29] Ma Z L, Xu B Q. Banking Structure Competition, Construction of Unified Market and Export Quality of Manufacturing Industry,2023(04):1–9. doi: 10.13902/j.cnki.syyj.2023.04.010

[30] Martin R. Regional economic resilience, hysteresis and recessionary shocks[J]. Journal of economic geography, 2012, 12(1): 1–32.

[31] Rao P G, Yue H, Jiang G H. Economic policy uncertainty and firms’ investment[J]. The Journal of World Economy, 2017, 40(2): 27–51.

[32] Brandt Loren, Johannes Van Biesebroeck, and Yifan Zhang. "Creative accounting or creative destruction? Firm-level productivity growth in Chinese manufacturing." Journal of development economics 97.2 (2012): 339–351. doi: 10.1016/j.jdeveco.2011.02.002

[33] Guo J, Zhou L L. Trade Policy Uncertainty, Tariff Change and Enterprises Survival[J]. Journal of International Trade,2019(05):22–40.

[34] Greenland A, Ion M, Lopresti J. Policy uncertainty and the margins of trade[J]. Manuscript, Elon University, University of Arizona, and The College of William and Mary, 2014.

[35] Wei Y Y, Liu H D. Study on the Influence of Economic Policy Uncertainty on the Margins of Exports—Theory and Empirical Evidence from China and Its Trading Partners[J]. International Business,2017(01):28–39.

[36] Bin Sheng, Mao Q L. Trade opening, domestic market integration and China’s interprovincial economic growth: 1985–2008[J]. The Journal of World Economy, 2011(11):44–66.

[37] Liu H D, Wu Q Y, Li W Y. Market-oriented Transition and Export Sophistication: A Perspective Based on Regional Market Integration[J]. Journal of International Trade, 2013(05):32–44.

[38] Liu X H. Market Fragmentation and Quality of Export Products—Evidence from Chinese Manufacturing Enterprises[J]. Journal of International Trade, 2020(11):30–44.

[39] Poncet S. Measuring Chinese domestic and international integration[J]. China Economic Review, 2003, 14(1): 1–21.

[40] Zhang J, Zhang P L, Huang T. Does domestic market segmentation push Chinese firms’ exporting[J]. Economic Research Journal, 2010, 8: 29–41.

[41] Gao Y. Export Firms and Integration of Domestic Market[J]. Journal of International Trade, 2016(12):142–154. doi: 10.13510/j.cnki.jit.2016.12.013

[42] Qiang Y C, Yang H Y. Market Integration, Spatial Spillover and Regional Export Quality Upgrading—Based on the Experience Analysis of Market Integration[J]. Journal of International Trade,2021(10):1–16.

[43] Melitz M J. The impact of trade on intra‐industry reallocations and aggregate industry productivity[J]. Econometrica, 2003, 71(6): 1695–1725.

[44] Baker S R, Bloom N, Davis S J. Measuring economic policy uncertainty[J]. The quarterly journal of economics, 2016, 131(4): 1593–1636.

[45] Yang R D, Study on the total factor productivity of Chinese manufacturing enterprises[J]. Economic Research Journal, 2015,50(02):61–74.

[46] Mayer T, Melitz M J, Ottaviano G I P. Market size, competition, and the product mix of exporters[J]. American Economic Review, 2014, 104(2): 495–536.

[47] Melitz M J, Ottaviano G I P. Market size, trade, and productivity[J]. The review of economic studies, 2008, 75(1): 295–316.

[48] Zhao Y Q, Ke S Z. Market Segmentation, Export Threshold of Productivity, and “Made in China”[J]. The Journal of World Economy,2016,39(09):74–98.

[49] Kroszner R S, Laeven L, Klingebiel D. Banking crises, financial dependence, and growth[J]. Journal of financial Economics, 2007, 84(1): 187–228.

